# Older adults and non-response to rabies post-exposure prophylaxis: Challenges and approaches

**DOI:** 10.14745/ccdr.v49i06a05

**Published:** 2023-06-01

**Authors:** Reed Morrison, Cindy Nguyen, Monir Taha, Robin SL Taylor

**Affiliations:** 1 Ottawa Public Health, Ottawa, ON; 2 Public Health and Preventive Medicine program, University of Ottawa, Ottawa, ON

**Keywords:** rabies, post-exposure prophylaxis, rabies vaccine, antibody response

## Abstract

Rabies vaccines are highly effective and immunogenic in most populations, including when used as rabies post-exposure prophylaxis (RPEP); however, there is mounting evidence that the immune response to rabies vaccines, though predicted to be adequate, may be lower in older adults. Despite this, there are no specific recommendations in Canadian guidance to monitor the serological response of older adults following RPEP. Furthermore, while Canadian guidance recommends the intramuscular route for RPEP vaccination, there is good evidence supporting the immunogenicity, effectiveness and safety of RPEP vaccination using the intradermal route. We present a case of an 87-year-old male with rabies exposure who failed to respond to two series of RPEP with intramuscular rabies vaccination but responded to a third series using intradermal vaccine administration and provide reasoning for subsequent management. This case is brought forward to prompt discussion and research as to the utility of completing serology in older adults receiving RPEP as well as vaccination strategies, including route of administration, in those who do not respond to an initial course of RPEP vaccination.

## Introduction

Public health professionals and clinicians in primary and emergency care commonly engage in risk assessment and management of potential rabies exposures. In Ottawa alone, a city of approximately 1 million persons, 1,305 rabies investigations were completed by Ottawa Public Health (OPH) in 2019, which resulted in 227 recommendations for rabies post-exposure prophylaxis (RPEP). Despite the regularity of managing potential rabies exposures, little attention is given to more complex cases such as RPEP non-responders. We present a case of an 87-year-old male with rabies exposure who failed to respond to two series of RPEP and provide reasoning for subsequent management.

Human rabies is rare in Ontario, with the last domestic case occurring in 1967. However, due to continued circulation in wild animal reservoirs and the near-certain mortality of the disease, rabies remains an important public health concern. Rabies disease is caused by viruses of the *Lyssavirus* genus, of which rabies virus (RABV) is the type species. In Canada, wild mammals such as bats, skunks, racoons and foxes are the reservoirs of RABV. RABV can be transmitted to humans through the saliva of an infected animal. While this is most often due to a bite, exposure of non-intact skin or mucosal surfaces to rabies virus-containing saliva can also result in infection ((1)).

Symptoms develop following an incubation period of 3–8 weeks, although this may be as short as a few days or as long as several years. Once symptoms develop, a prodrome characterized by apprehension, excitability, headache, non-specific sensory changes and fever can last between two and 10 days. The disease then progresses to an acute neurological phase consisting of encephalomyelitis and cardiac failure that is nearly always fatal, even when medical care is provided ((1)).

## Case

The patient was an 87-year-old male living in a long-term care home (LTCH). Informed consent to share his case was provided by his substitute decision maker. His comorbidities included type 2 diabetes mellitus, Parkinson’s disease, dementia, sinus bradycardia, orthostatic hypotension and malnutrition as evidenced by low serum protein and knowledge of his dietary intake. The patient was not previously vaccinated against rabies.

During an evening in the spring of 2021, the patient was observed by staff to come into direct (skin) contact with a bat that had entered the LTCH. The patient informed staff that the bat was in contact with his left index finger. Staff noted small wounds on the skin in that area with no visible bite marks. Immediately following exposure to the bat, the patient’s wounds were cleaned with saline.

The bat was captured by LTCH staff and delivered to animal control for rabies testing. Ottawa Public Health was notified of the exposure at this time. A rabies risk assessment was performed in consultation with staff at the LTCH. Due to the high-risk exposure, it was recommended that the patient receive RPEP consisting of body-weight-based rabies immunoglobulin (RabIg) and a vaccination series. Ottawa Public Health was notified three days later that the bat had tested positive for RABV, which meant completing the full RPEP series was indicated.

### Post-exposure prophylaxis

Rabies post-exposure prophylaxis is provided to individuals following a confirmed rabies exposure. The Canadian Immunization Guide (CIG) provides guidance regarding the risk assessment for rabies following exposure to potentially rabid animals. Post-exposure prophylaxis or testing of a bat is generally recommended after direct contact with a bat because it is very difficult to ensure that a bite did not take place ((2)).

Rabies post-exposure prophylaxis for unimmunized persons consists of providing immediate passive immunity immunization through RabIg and eliciting active immunity immunization through a rabies vaccination series. Immunocompetent individuals are recommended to receive a series of four 1.0 mL intramuscular (IM) doses of an approved vaccine on days 0, 3, 7, and 14. Immunocompromised individuals receive an additional dose on day 28. There are two vaccine preparations approved for use in Canada: the inactivated human diploid cell rabies vaccine (HDCV) and the inactivated purified chick embryo cell rabies vaccine (PCECV) ((2)).

The IM route is the only recommended route of vaccine administration for RPEP in the CIG ((2)). Conversely, the World Health Organization recommends either the IM or intradermal (ID) route for RPEP, noting that many clinical trials have confirmed immunogenicity, effectiveness, and safety of RPEP using the ID route ((3)). A recent statement by the Ontario Immunization Advisory Committee notes that, within Canada, British Columbia and Alberta are the only provinces to have implemented a recommendation for the ID route of RPEP. The statement recommends that the “rabies vaccine for post-exposure prophylaxis should continue to be provided using the IM route of administration in Ontario at this time.” While recognizing evidence of safety and effectiveness of the ID route, the statement highlights epidemiologic, logistical, and cost considerations for their recommendation ((4)).

### Case, revisited

Following convention, the date RPEP begins is “day 0”, and all events that follow are calculated in days following RPEP initiation. All vaccination doses, routes and serology results are summarized in **Table 1**.

**Table 1:** Summary of vaccination and serology results

Date	Day #/Series	Product	Administration route	Dose	Serology results^a^
2021-05-24	Day 0	Rabies Immunoglobulin (Human) (KamRAB^TM^)	IM left deltoid	15% of total^b^	N/A
2021-05-24	Day 0/Series 1	PCECV (RabAvert**^®^)**	IM right deltoid	1 mL	N/A
2021-05-25	Day 1	Rabies Immunoglobulin (Human) (KamRAB)	Wound infiltration left index finger and IM left deltoid	85% of total^b^	N/A
2021-05-27	Day 3/Series 1	PCECV (RabAvert**)**	IM right deltoid	1 mL	N/A
2021-05-31	Day 7/Series 1	PCECV (RabAvert**)**	IM right deltoid	1 mL	N/A
2021-06-07	Day 14/Series 1	PCECV (RabAvert**)**	IM right deltoid	1 mL	0.0 IU/mL
2021-06-18	Day 25	N/A	N/A	N/A	0.0 IU/mL
2021-06-22	Day 0/Series 2	HDCV (IMOVAX**^®^ Rabies)**	IM left deltoid	1 mL	N/A
2021-06-25	Day 3/Series 2	HDCV (IMOVAX **Rabies)**	IM right deltoid	1 mL	N/A
2021-06-29	Day 7/Series 2	HDCV (IMOVAX **Rabies)**	IM right deltoid	1 mL	N/A
2021-07-06	Day 14/Series 2	HDCV (IMOVAX **Rabies)**	IM right deltoid	1 mL	N/A
2021-07-09	Day 17	N/A	N/A	N/A	0.0 IU/mL
2021-07-19	Day 27	N/A	N/A	N/A	0.0 IU/mL
2021-07-20	Day 28/Series 2	HDCV (IMOVAX **Rabies)**	IM right deltoid	1 mL	N/A
2021-08-07	Day 46	N/A	N/A	N/A	0.0 IU/mL
2021-08-12	Day 0/Series 3	HDCV (IMOVAX **Rabies)**	ID right and left deltoid areas	0.1 mL x 2=0.2 mL	N/A
2021-08-15	Day 3/Series 3	HDCV (IMOVAX **Rabies)**	ID right and left deltoid areas	0.1 mL x 2=0.2 mL	N/A
2021-08-19	Day 7/Series 3	HDCV (IMOVAX **Rabies)**	ID right and left deltoid areas	0.1 mL x 2=0.2 mL	N/A
2021-09-03	Day 22	N/A	N/A	N/A	1.28 IU/mL

Abbreviations: HDCV, human diploid cell rabies vaccine; ID, intradermal; IM, intramuscular; N/A, not applicable; PCECV, purified chick embryo cell rabies vaccine

^a^ Listed by the day of blood draw

^b^ Dose in mL is not provided to protect personal health information

The patient received 15% of the total body weight-based RabIg (KamRAB^TM^ human rabies immunoglobulin) dose in the left deltoid the morning following exposure (day 0) and the remaining 85% in the left index finger and left deltoid on day 1 (an insufficient dose of RabIg provided to the patient on day 0 necessitated a further dose the next day to achieve an adequate weight-based dosage). The patient received a total RabIg dose of 20 IU/kg as per CIG recommendations.

In accordance with guidelines, a four-dose series of PCECV (RabAvert^®^) vaccine was given IM on days 0, 3, 7, and 14 in the right deltoid.

Ottawa Public Health routinely recommends serology 7–14 days following an RPEP series for those 70 years of age and older due to a lack of compelling literature evidence demonstrating adequate serological response in that age group. As such, due to the patient’s age, blood for serology was drawn 11 days after the fourth vaccine dose. Results showed no detectable rabies antibodies (Table 1).

Given the initial non-response, and in accordance with CIG recommendations, a second series consisting of five doses of HDCV (IMOVAX^®^ Rabies) was delivered IM on newly established days 0, 3, 7, 14, and 28. Multiple repeated serological determinations showed no detectable antibodies (Table 1). The inadequate serological response was hypothesized to be due primarily to the patient’s advanced age with possible contribution of malnutrition (*personal communication with treating physician, n.d.*). Ottawa Public Health consulted the respective vaccine manufacturers and no deficiencies in effectiveness with the associated lot numbers were identified. Similarly, consultation with laboratory colleagues revealed a high level of confidence in the results. A cold chain failure is an unlikely contributing factor as OPH supplied the vaccine directly to the LTCH and there were no noted gaps in the cold chain. Lastly, administration error is also unlikely as OPH provides teaching on administration of rabies vaccines as part of standard practice.

Based on literature review and in consultation with expert colleagues, OPH and the patient’s substitute decision maker decided to proceed with a third series of RPEP vaccination using the ID route. The patient received 0.1 mL ID of HDCV (IMOVAX Rabies) in the skin overlying each of the right and left deltoids (total 0.2 mL) on newly established days 0, 3, and 7. Blood for serology drawn 15 days after the final vaccine dose was reactive at 1.28 IU/mL.

## Effectiveness and immunogenicity

The rabies vaccine is highly effective and immunogenic in most populations. In the context of RPEP, evidence suggests that close to 100% of healthy individuals will have an adequate antibody response within 14–30 days of finishing a vaccination series ((2,5–7)). Real-world treatment failures are extremely rare and are often due to deviations from accepted RPEP protocols ((8)). There have been no documented RPEP failures in Canada ((2)).

Despite overall high immunogenicity, there are some populations that are more likely to demonstrate lower antibody responses. Those with immunosuppression, and particularly those with very low CD4 counts, are at the highest risk of not seroconverting following vaccination ((9)). There is also mounting evidence that the immune response to rabies vaccines may be lower in older adults ((9)). For example, a recent meta-analysis found that adults older than 50 years of age had lower maximal mean antibody titres following an RPEP regimen. Although maximal mean titres were protective (higher than 0.5 IU/mL), there were lower rates of seroconversion in those older than 50 years of age when compared to those younger than 50 years ((7)). Similarly, pre-exposure prophylaxis studies ((10,11)) have found an age-based gradient of decreasing immunogenicity and seroconversion. Additional studies on pre-booster antibody titres have also found an age-based gradient ((12)).

Unfortunately, there is a research gap regarding the immunogenicity of the rabies vaccine in adults older than 70 years of age. A robust literature search strategy conducted for this article identified a single case series that separately analyzed the data of individuals older than 70 years of age. This study compared the antibody titres of different age groups, including 10 individuals older than 70 years of age, and did not find an age-based difference ((13)).

### Monitoring rabies post-exposure prophylaxis response

Because the rabies vaccine is highly immunogenic and effective in most populations, there are very few circumstances when the CIG recommends serology following RPEP. In immunocompetent persons, serology is recommended 7–14 days after completing an RPEP series only when there has been “substantial deviation” from the recommended schedule or a non-recommended vaccine has been used ((2)).

The CIG recommends serology 7–14 days after a five-dose RPEP series in immunocompromised persons ((2)). Despite evidence suggesting a lower immune response in older adults to rabies vaccines, older age is not considered an immunocompromising risk factor in the CIG; therefore, there are no specific recommendations for monitoring serology in older adults following RPEP. While this is consistent with some pieces of guidance including that of the World Health Organization ((3)), French guidance ((14)) does suggest completing serology following a course of RPEP in older adults.

If there has been an inadequate antibody response to vaccination, a second series of vaccination is recommended along with further serological testing. The CIG does not provide further vaccination guidance if the second RPEP vaccination series does not produce an adequate antibody response, stating instead that “some immunocompromised people may never mount an appropriate immune response” ((2)). A review of the published and grey literature also could not identify any relevant guidance documents or statements that provide direction following an inadequate serological response to two complete RPEP series.

The British HIV Association ((15)) provides suggestions for patients with human immunodeficiency virus (HIV) who fail to seroconvert after an initial RPEP regimen, which are potentially instructive for other non-responders (including multiple non-responders after multiple vaccine series). They propose that these individuals should be offered double-dose and/or more frequent vaccine doses and should be considered for a combination of ID and subcutaneous routes during a subsequent RPEP vaccination series. Similar strategies have also been used in HIV-negative immunocompromised patients ((16)).

## Discussion

This case is unique as it is the first published instance of an individual demonstrating serological response to the ID route of vaccination for RPEP following a non-response to the IM route. This is important as very little is known about changing the route of administration of RPEP vaccination. A recent systematic review identified just two studies on the topic ((17)). Based on the limited evidence, the authors concluded that changing the route of administration either during a course of RPEP or between a pre-exposure prophylaxis series and a RPEP series was a safe and effective way to achieve a serological response. The review did not identify any previous research on changing routes of administration between RPEP vaccination series, as this case discusses.

This case is also unique in demonstrating an adequate serological response after a third series of RPEP vaccination. Previous research has found that those who do not respond to an initial course of RPEP almost uniformly respond following additional doses ((16,18)). Rarely, chronic non-responders, usually with severe immune suppression, have been identified and do not seroconvert regardless of re-vaccination strategy ((2,19)).

The patient’s adequate serological response following ID vaccination may reflect the skin’s important role in the immune system and specifically the higher concentration of antigen-presenting cells in the skin ((8)). It may also have been a function of a higher cumulative total vaccine dose. It is unclear if this patient would have seroconverted sooner had we attempted other strategies during the second round of RPEP such as administration of vaccine using the ID route, “double dosing” or more frequent dosing, although these strategies are not addressed in the CIG.

## Conclusion

Given how frequently potential rabies exposures are managed, it is important for clinicians and public health practitioners to be aware of approaches to complex cases such as RPEP non-responders. This case prompts three considerations for public health practice. First, due to the life-threatening nature of the disease, we believe it is medically prudent to complete serology in adults 70 years of age and older following each RPEP series until new research can more confidently describe the nature of the immune response of older adults to rabies vaccination. Second, we contend that clinicians may consider the circumstances under which a different route of vaccine administration would benefit those who do not respond to an initial RPEP series. Lastly, given that neither the CIG nor other guidance documents identified during a literature review provide case management advice for individuals who fail to seroconvert following two RPEP vaccination series, we recommend that further research and guidelines be developed to address this gap.

## References

[1] Ministry of Health. Ontario Public Health Standards: Requirements for Programs, Services and Accountability, Infectious Disease Protocol, Appendix 1: Case Definitions and Disease-Specific Information, Disease: Rabies. Toronto, ON: Ministry of Health, 2022. https://www.health.gov.on.ca/en/pro/programs/publichealth/oph_standards/docs/rabies_chapter.pdf

[2] Public Health Agency of Canada. Rabies vaccine: Canadian Immunization Guide. Ottawa, ON: PHAC; 2015. https://www.canada.ca/en/public-health/services/publications/healthy-living/canadian-immunization-guide-part-4-active-vaccines/page-18-rabies-vaccine.html

[3] World Health Organization. Rabies vaccines: WHO position paper, April 2018 - Recommendations. Vaccine 2018;36(37):5500–3. 10.1016/j.vaccine.2018.06.061 30107991

[4] Public Health Ontario; Ontario Immunization Advisory Committee. Use of the intradermal route for rabies vaccine post-exposure prophylaxis in Ontario. Toronto, ON: Government of Ontario; 2022. https://www.publichealthontario.ca/-/media/Documents/O/2022/oiac-intradermal-route-rabies-vaccine-post-exposure.pdf?rev=e63870749eea42559877c60990723300&sc_lang=en

[5] Denis M, Knezevic I, Wilde H, Hemachudha T, Briggs D, Knopf L. An overview of the immunogenicity and effectiveness of current human rabies vaccines administered by intradermal route. Vaccine 2019;37 Suppl 1:A99–106. 10.1016/j.vaccine.2018.11.072 30551985

[6] Li R, Li Y, Wen S, Wen H, Nong Y, Mo Z, Xie F, Pellegrini M. Immunogenicity and safety of purified chick-embryo cell rabies vaccine under Zagreb 2-1-1 or 5-dose Essen regimen in Chinese children 6 to 17 years old and adults over 50 years: a randomized open-label study. Hum Vaccin Immunother 2015;11(2):435–42. 10.4161/21645515.2014.994460 25692350 PMC4514244

[7] Xu C, Lau CL, Clark J, Rafferty AC, Mills DJ, Ramsey L, Gilbert B, Doi SA, Furuya-Kanamori L. Immunogenicity after pre- and post-exposure rabies vaccination: A systematic review and dose-response meta-analysis. Vaccine 2021;39(7):1044–50. 10.1016/j.vaccine.2021.01.023 33478786

[8] Wilde H. Failures of post-exposure rabies prophylaxis. Vaccine 2007;25(44):7605–9. 10.1016/j.vaccine.2007.08.054 17905484

[9] World Health Organization. The immunological basis for immunization series: module 17: rabies. Geneva (CH): WHO; 2017. https://apps.who.int/iris/handle/10665/259511

[10] Furuya-Kanamori L, Ramsey L, Manson M, Gilbert B, Lau CL. Intradermal rabies pre-exposure vaccination schedules in older travellers: comparison of immunogenicity post-primary course and post-booster. J Travel Med 2020;27(7):taaa006. 10.1093/jtm/taaa006 31943042

[11] Mills DJ, Lau CL, Fearnley EJ, Weinstein P. The immunogenicity of a modified intradermal pre-exposure rabies vaccination schedule--a case series of 420 travelers. J Travel Med 2011;18(5):327–32. 10.1111/j.1708-8305.2011.00540.x 21896096

[12] Morris J, Crowcroft NS, Fooks AR, Brookes SM, Andrews N. Rabies antibody levels in bat handlers in the United Kingdom: immune response before and after purified chick embryo cell rabies booster vaccination. Hum Vaccin 2007;3(5):165–70. 10.4161/hv.3.5.4216 17786037

[13] Gautret P, Vu Hai V, Soavi MJ, Parola P, Brouqui P. [Influence of age on antibody titers following rabies post-exposure prophylaxis]. Pathol Biol (Paris) 2012;60(5):322–3. 10.1016/j.patbio.2011.09.001 22041176

[14] Direction générale de la Santé, Comité technique des vaccinations. Guide des vaccinations. Édition 2012. Saint-Denis: Inpes, coll. Varia, 2012: 488 p. http://affairesjuridiques.aphp.fr/textes/guide-des-vaccinations-direction-generale-de-la-sante-comite-technique-des-vaccinations-edition-2012-vaccination/

[15] Geretti AM, Brook G, Cameron C, Chadwick D, French N, Heyderman R, Ho A, Hunter M, Ladhani S, Lawton M, MacMahon E, McSorley J, Pozniak A, Rodger A. British HIV Association Guidelines on the Use of Vaccines in HIV-Positive Adults 2015. HIV Med 2016;17 Suppl 3:s2–81. 10.1111/hiv.12424 27568789

[16] Hay E, Derazon H, Bukish N, Scharf S, Rishpon S. Postexposure rabies prophylaxis in a patient with lymphoma. JAMA 2001;285(2):166–7. 10.1001/jama.285.2.166 11176806

[17] Kessels J, Tarantola A, Salahuddin N, Blumberg L, Knopf L. Rabies post-exposure prophylaxis: A systematic review on abridged vaccination schedules and the effect of changing administration routes during a single course. Vaccine 2019;37 Suppl 1:A107–17. 10.1016/j.vaccine.2019.01.041 30737043

[18] Uwanyiligira M, Landry P, Genton B, de Valliere S. Rabies postexposure prophylaxis in routine practice in view of the new Centers for Disease Control and Prevention and World Health Organization recommendations. Clin Infect Dis 2012;55(2):201–5. 10.1093/cid/cis384 22550115

[19] Wilde H. Editorial commentary: rabies postexposure vaccination: are antibody responses adequate? Clin Infect Dis 2012;55(2):206–8. 10.1093/cid/cis389 22550116

